# Experience Improves Spatial Foraging Performance in a Complex Environment in *Heliconius* Butterflies

**DOI:** 10.1002/ece3.74541

**Published:** 2026-10-27

**Authors:** P. A. Moura, M. Z. Cardoso, S. H. Montgomery

**Affiliations:** ^1^ Departamento de Ecologia Universidade Federal do Rio Grande do Norte Natal RN Brazil; ^2^ Department of Integrative Biology University of Texas at Austin Austin Texas USA; ^3^ Departamento de Ecologia, Instituto de Biologia Universidade Federal do Rio de Janeiro Rio de Janeiro RJ Brazil; ^4^ School of Biological Sciences University of Bristol Bristol UK; ^5^ Department of Zoology University of Cambridge Cambridge UK

**Keywords:** environmental complexity, foraging efficiency, insect navigation, pollen‐feeding butterflies

## Abstract

*Heliconius* butterflies rely on spatial memory to maintain trapline foraging routes, repeatedly visiting pollen resources along memorized paths. Increased foraging performance resulting from learning the spatial distribution of resources is thought to have driven the evolution of this specialized behavior. Here, we experimentally test whether prior experience in a spatially complex environment influences relative foraging performance in the pollen‐feeding butterfly *Heliconius erato* , a species with well‐documented behavioral, ecological and neuroanatomical specializations for spatial learning. We reared freshly eclosed adults in either an open cage or a cage that was matched for resource distribution but structurally divided to increase the spatial complexity experienced by the butterflies. We then tested them individually in a controlled foraging assay under familiar and unfamiliar environments. Butterflies reared in the spatially complex environment efficiently reached the feeder regardless of familiarity with the environment, whereas butterflies reared in the open environment showed strong context dependence, performing well only in the open cage. These results indicate that prior experience of a spatially complex environment supports greater foraging efficiency in *Heliconius* butterflies, highlighting environmental structure as a likely determinant of the ecological value of spatial learning in this group, with potential consequences for foraging behavior in natural environments.

## Graphical abstract

We experimentally tested whether prior experience in a spatially complex environment influences foraging performance in the pollen‐feeding butterfly *Heliconius erato* . Butterflies reared in a structurally complex environment efficiently reached a feeder under both familiar and unfamiliar conditions, whereas those reared in a simple environment performed well only in their familiar cage. These findings identify environmental structure as an important determinant of the ecological benefits of spatial learning in *Heliconius*.

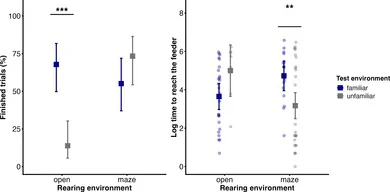

## 1 Introduction

Environmental complexity can have long‐term impacts, affecting brain plasticity and learning performance in both vertebrates (van Praag et al. 2000; Brown et al. 2003; Rodewald et al. 2011; Clarin et al. 2013) and invertebrates (Carducci and Jakob 2000; Scotto‐Lomassese et al. 2000; Cayre et al. 2007; Díaz‐Fleischer et al. 2009; Carle 2021). More complex environments can favor behavioral adaptations that increase foraging efficiency, learning speed, and problem solving, as shown in fishes (Brown et al. 2003; Rodewald et al. 2011), spiders (Carducci and Jakob 2000), and insects (Scotto‐Lomassese et al. 2000; Cayre et al. 2007; Díaz‐Fleischer et al. 2009; Carle 2021). This may occur in a developmentally plastic manner, but may also lead to the emergence of specialized foraging behaviors that depend on learning and memory. For example, trapline foraging in pollinators involves learned optimization of repeatable routes among resource patches (Thomson et al. 1982; Ohashi and Thomson 2009; Lihoreau et al. 2011). Trapline foraging has been demonstrated in orchid bees (Janzen 1971), bumblebees (Heinrich 1976), honey bees (Buatois and Lihoreau 2016) and hornets (Lacombrade et al. 2025), and involves establishing preferable foraging routes by repeatedly visiting a series of food locations in a predictable order, thereby improving the efficiency of finding and exploiting food resources (Thomson et al. 1982; Janzen 1971). For instance, in bumblebees, experience of spatial foraging is associated with a refinement of foraging flight paths, suggesting improved efficiency (Heinrich 1976), while retaining the flexibility to adapt to and incorporate changes in resource distribution in a complex environment (Ohashi et al. 2007; Lihoreau et al. 2012).

Compared to Hymenoptera, *Heliconius* butterflies display a relatively recent transition to a specialized foraging strategy that may reflect an adaptation to complex resource distributions in the environment favoring investment in spatial memory. These butterflies are particularly notable for their unique dietary adaptation, actively collecting and digesting pollen grains from a relatively restricted set of plants (Gilbert 1972; Boggs et al. 1981; Young and Montgomery 2020), providing adults with a source of essential amino acids (O'Brien et al. 2003) associated with increased longevity (Ehrlich and Gilbert 1973), delayed reproductive senescence (Dunlap‐Pianka 1979), chemical communication and chemical defense (Gilbert 1991; Cardoso and Gilbert 2013; Pinheiro de Castro et al. 2020). To support this feeding strategy, *Heliconius* establish foraging routes in which specific plants are revisited with spatial and temporal regularity, a behavior known as trapline foraging (Young and Montgomery 2020; Gilbert 1975). These routes are initiated from stable nocturnal roosting sites, to which individuals return consistently and faithfully (Jones 1930; Waller and Gilbert 1982; Mallet 1986; Finkbeiner et al. 2012; Finkbeiner 2014). From these roosts, butterflies depart each day to repeatedly visit a restricted set of pollen plants, a behavior that requires robust spatial memory. Experimental evidence also supports these observations from the field, demonstrating that *Heliconius* butterflies can learn and remember the locations of food sources across varying spatial scales (Moura, Young, et al. 2023; Moura et al. 2024), which is crucial for their foraging efficiency. They are also able to quickly orient towards, and return to, their home sites when translocated hundreds of meters to unknown territory, showing remarkable site fidelity (Moura et al. 2022).

Comparative neuroanatomical work has further shown that *Heliconius* butterflies have significantly enlarged, visually specialized mushroom bodies, brain regions associated with sensory integration and memory, particularly in foraging contexts (Montgomery et al. 2016; Couto et al. 2023; Young and Montgomery 2023). *Heliconius* also exhibit high degrees of plasticity in the mushroom bodies, as a function of age and environmental experience (Montgomery et al. 2016; Hebberecht et al. 2023; Montgomery and Merrill 2017; Young, Alcalde Anton, et al. 2024), with captive‐reared butterflies having smaller mushroom bodies than wild‐caught butterflies, unless reared in large seminatural enclosures (Hebberecht et al. 2023). This pattern is consistent with the hypothesis that mushroom body neural plasticity supports flexible foraging behaviors that are responsive to environmental complexity, such that learning and memory invoke neural change that facilitates improvements in foraging efficiency. Indeed, *Heliconius* butterflies do exhibit a remarkable range of cognitive abilities, including spatial perception (Moura, Young, et al. 2023; Moura et al. 2022; Cardoso et al. 2024), associative learning (Young, Alcalde Anton, et al. 2024; Toure et al. 2020; Young, Melo‐Flórez, et al. 2024), and visual memory (Jones 1930; Moura et al. 2024), despite their relatively small brains (Montgomery et al. 2016; Sivinski 1989). However, despite evidence of cognitive specialization in *Heliconius*, few studies (Hebberecht et al. 2023) have tested their spatial learning abilities or whether learning improves foraging efficiency in structured environments.

Here, we test the hypothesis that prior experience with a spatially complex environment improves foraging efficiency under spatially structured than under simple environmental conditions in *Heliconius* butterflies. We reared adult *H. erato* butterflies in either complex or simple spatial enclosures and subsequently assessed their performance in both environments. Because individuals were tested only in the two rearing environments, our design tests performance across familiar and unfamiliar contexts differing in spatial complexity, rather than the transfer of spatial experience to a completely novel complex environment. However, as *Heliconius* form stable home ranges, learning in the context of a consistent, familiar habitat is central to their foraging strategy (Jones 1930; Waller and Gilbert 1982; Mallet 1986; Finkbeiner et al. 2012; Finkbeiner 2014). Our approach builds on a previous study (Hebberecht et al. 2023) by focusing on a different *Heliconius* species from a distinct lineage to test whether these effects replicate and generalize across the genus, while also testing a larger number of individuals and conducting more extensive behavioral testing. We predicted that prior experience with the complex environment would be associated with greater foraging efficiency in that condition, reflecting an increased benefit of spatial memory in more complex spatial conditions.

## 2 Materials and Methods

### 2.1 Experimental Subjects and Environments

Experimental subjects originated from first‐generation insectary‐reared stock populations of *Heliconius erato* *phyllis*, descended from multiple wild‐caught female butterflies collected at different areas in Mata do Jiqui, Natal, Brazil (5°55′39″ S, 35°10′59″ W). We maintained stock populations in an outdoor flight cage (3 × 3 × 2 m) containing host plants. Experiments were conducted in 2020 during the COVID‐19 lockdown, resulting in constraints on the space available for experimental setup.

Individuals used in the experiment consisted of freshly eclosed adults that were assigned to one of two experimental outdoor cage environments (3 × 3.5 × 2.5 m) differing in environmental complexity (Figure 1). In both environments, butterflies had access to a host plant species (*Passiflora misera*) and to a rewarding artificial feeder made from red EVA foam sheets and yellow plastic straws and placed at 1 m above the ground. Feeders contained a 20% sugar solution supplemented with bee pollen to provide amino acids. Ground cover consisted of natural grass and visual surroundings of the two cages were similar, which helped minimize differences in factors such as light exposure and orientation. Because each treatment was represented by a single cage, however, cage‐specific effects cannot be fully separated from treatment effects within the present experiment. Nevertheless, the consistency of the observed pattern with our previous experiment using the same experimental treatments provides additional support that the differences observed here are associated with environmental complexity rather than idiosyncratic differences between cages. All butterflies were uniquely labeled using a nontoxic ink on the ventral surface of their forewings.

**FIGURE 1:**
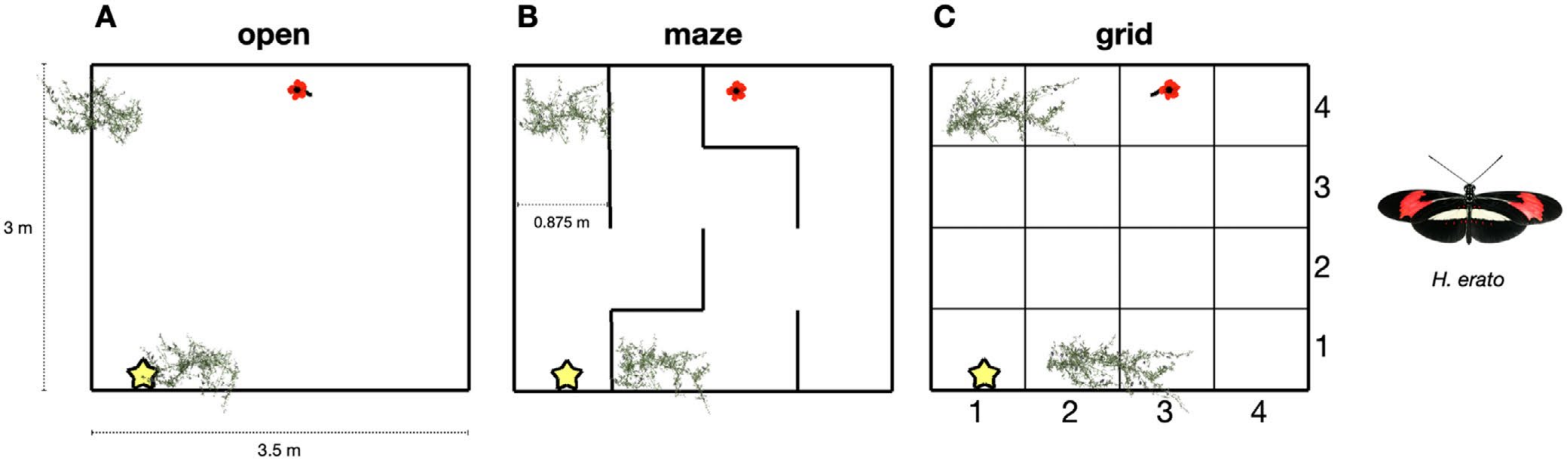
Experimental environment configurations and spatial reference system. (A) Open environment with no added spatial complexity. (B) Maze environment with increased spatial complexity. (C) Analytical grid system used to record butterfly positions (3 × 3.5 m), with cells measuring 0.75 × 0.875 m. Red flowers indicate feeders, green plants indicate host plants, and yellow stars indicate release points for the foraging assay.

Each environment presented a specific spatial challenge: an open environment (open) or a maze‐like environment (maze). To create the *maze* configuration, we installed internal walls made of translucent gray mesh netting with ~0.875 m wide corridors that partially obstructed visibility and constrained movement and access to resources (Figure 1B). In contrast, the *open* environment contained no internal partitions, providing unobstructed visibility and direct paths between resources (Figure 1A). Resource placement was identical in both environments, with the presence or absence of internal walls as the sole experimental difference.

Freshly‐eclosed butterflies were randomly assigned to one of the two environmental conditions, providing no basis for initial differences in naïve performance between groups. Differences observed in the final behavioral trials could therefore be interpreted as effects of experience in either a subdivided or open enclosure. Butterflies were allowed to fly freely within their respective environments for 15–36 days depending on their eclosion time and the start day in the experiment. Butterflies added on the first day remained in their assigned environment for the full 36‐day period, whereas those introduced on day 21 remained 15 days. A total of 80 butterflies were tested across the two cages, with individuals distributed between the maze (*n* = 38) and open environments (*n* = 42). We consider the influence of social learning unlikely in this task, given that previous experiments under comparable conditions found no evidence of social information in *H. erato* (Moura, Cardoso, and Montgomery 2023).

### 2.2 Foraging Assay

At the end of the 36‐day period, we conducted a foraging assay to test the spatial performance of individual butterflies reared under different environmental complexities. All butterflies were gently collected at night in their roosting positions and placed overnight in translucent pop‐up cages, grouped according to their home environment and kept in the stock population flight cage.

The following morning, butterflies were released individually into both environments through a small door (Figure 1, yellow star). Each individual was first released in the unfamiliar environment, subsequently recaptured and placed in a translucent pop‐up cage for 1 h, and then tested in the familiar environment. Butterflies were observed for 5 min per trial or until they reached the feeder, which was clean and unrewarded during the assay to remove olfactory cues. The duration of the foraging assay was based on previous experiments conducted under similar conditions, as butterflies typically lose interest after approximately 5 min (Hebberecht et al. 2023). For each trial, we recorded whether the butterfly reached the feeder, the latency to reach the feeder (in seconds), and the individual's final position within the cage environment using an analytical 4 × 4 grid system (16 spatial cells), with positions ranging from A1 to D4 (Figure 1C).

### 2.3 Statistical Analysis

To test effects of “test condition” (familiar vs. unfamiliar) and “environment” (maze vs. open) on task performance, we analyzed two response variables: (i) trial completion and (ii) time to reach the feeder. Trial completion (finished vs. not finished) was analyzed using a generalized linear mixed‐effects model (GLMM) in R (R Core Team 2026) with the glmer function from the package lme4 v 1.1–37 (Bates et al. 2015). All models were fitted with a binomial error distribution and logit link and included individual identity as a random effect to account for repeated measures. “Test condition,” “environment,” their interaction, and “age” were included as fixed effects. Sex had no effect in any comparison and was therefore excluded from the final models. Model significance was evaluated using likelihood‐ratio tests. Post hoc pairwise comparisons were performed using estimated marginal means with Tukey adjustment. Model assumptions were assessed using simulated residuals implemented in the package DHARMa v 0.4.7 (Hartig 2024). For butterflies that successfully reached the feeder, time to reach the feeder was analyzed using a linear model including “test condition,” “environment,” their interaction, and “age” as fixed effects. Because initial models violated assumptions of normality and homoscedasticity, time was log‐transformed prior to analysis. Model assumptions were verified using simulated residuals, and post hoc comparisons were performed using estimated marginal means. Additionally, heatmaps were generated to visualize final spatial positions for all butterflies, with counts of observations calculated for each cell of a 4 × 4 spatial grid for each test environment and rearing environment combination.

## 3 Results

We predicted that familiarity with the environment would increase a butterfly's ability to reach the feeder within 5 min, such that individuals would successfully complete the task when tested in their home environment. In the unfamiliar condition, expectations differed between environment types. Owing to the simplified spatial structure of the open environment, we predicted that individuals reared in the maze environment would successfully locate the feeder when tested in the open environment within the allotted time. In contrast, given the increased spatial complexity of the maze environment, we predicted that individuals reared in the open environment would be less likely to reach the feeder when tested in the maze environment.

We found that the probability of reaching the feeder was determined by a significant interaction between “environment” (open/maze) and “test condition” (familiar/unfamiliar) (*β* = −3.39 ± 0.89, *z* = −3.80, *p* = 0.0001). Age also had an additional negative effect (*β* = −0.11 ± 0.04, *z* = −2.56, *p* = 0.010), with older individuals reaching the feeder less often across test environments and rearing environments (Figure S1). Post hoc comparisons showed no effect of familiarity in butterflies reared in the maze environment (EMM = −0.806 ± 0.536, *z* = −1.50, *p* = 0.133), where EMM represents the difference between estimated marginal means (familiar–unfamiliar), with the majority of individuals reaching the feeder in both familiar (55%; 21/38) and unfamiliar (71%; 27/38) conditions (Figure 2A). By contrast, unfamiliarity significantly reduced the probability of reaching the feeder for butterflies reared in the open environment (EMM = 2.574 ± 0.659, *z* = 3.90, *p* < 0.0001), with 64% (27/42) of individuals reaching the feeder under familiar conditions, compared with only 17% (7/42) under unfamiliar conditions (Figure 2A). Likewise, “environment” had no effect under familiar conditions (EMM = −0.540 ± 0.531, *z* = −1.02, *p* = 0.309), but strongly influenced performance under unfamiliar conditions, with butterflies reared in the open environment being less likely to reach the feeder (EMM = 2.840 ± 0.711, *z* = 4.00, *p* < 0.0001).

**FIGURE 2:**
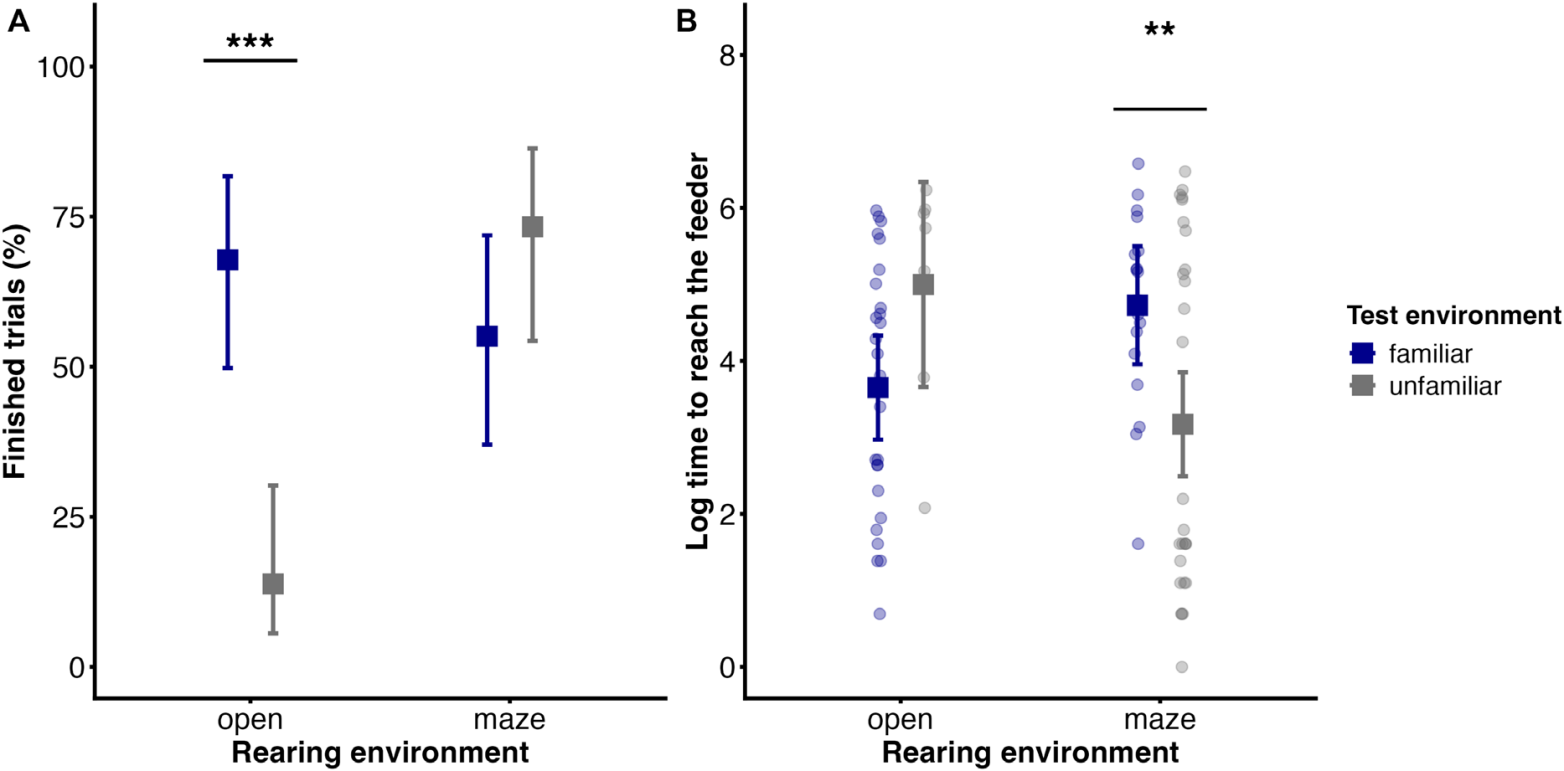
Foraging performance across experimental conditions in the 5‐min foraging assay. (A) Percentage of individuals reaching the feeder (*n* = 42 in the open environment; *n* = 38 in the maze environment; total *n* = 80). (B) Log‐transformed time to reach the feeder (*n* = 27 in open familiar; *n* = 7 in open unfamiliar; *n* = 21 in maze familiar; *n* = 27 in maze unfamiliar). Boxes and whiskers represent means ±95% CI. In all panels, *maze* denotes the environment with internal partitions, and *open* denotes the environment without internal partitions. *Familiar* indicates individuals tested in their home environment, whereas *unfamiliar* indicates individuals tested in the alternative environment. **p < 0.01, ***p < 0.001.

Concerning time taken to reach the feeder, we predicted that increased spatial complexity would be detrimental to butterflies' performance, such that individuals successfully completing the task would do so more rapidly in the open environment than in the maze environment, regardless of familiarity with the environment. In fact, time to reach the feeder among successful individuals was influenced by a significant interaction between “environment” (open/maze) and “test condition” (familiar/unfamiliar) (*F*_1,77_ = 9.94, *p* = 0.002). Post hoc comparisons revealed that, in the maze environment, butterflies reached the feeder significantly faster in the open environment than in their home environment (EMM = 1.56 ± 0.52, *t*_77_ = −3.00, *p* = 0.004; Figure 2B). In contrast, for butterflies reared in the open environment, no significant difference was detected between familiar and unfamiliar conditions (EMM = −1.35 ± 0.76, *t*_77_ = −1.78, *p* = 0.079; Figure 2B), however, this result may reflect the smaller number of individuals from the open environment that completed the task under unfamiliar conditions (17% (7/42) vs. 64% (27/42)). Comparisons between environment types showed that, under familiar and unfamiliar conditions, butterflies reached the feeder more rapidly in the open environment than in the maze (EMM = 1.08 ± 0.52, *t*_77_ = 2.08, *p* = 0.041; EMM = −1.83 ± 0.76, *t*_77_ = −2.41, *p* = 0.018, respectively). Age did not have any effect on the time taken for the butterflies to reach the feeder.

Concerning individuals' final spatial positions at the end of the foraging assay, butterflies reared in the maze environment clustered strongly around the feeder location (Figure 3, left panels; red asterisk) under both familiar and unfamiliar test conditions, indicating consistent navigation towards the food source. In contrast, butterflies reared in the open environment clustered strongly around the feeder only under familiar conditions, whereas under unfamiliar conditions individuals were more spatially dispersed at the end of the assay (Figure 3, right panels).

**FIGURE 3:**
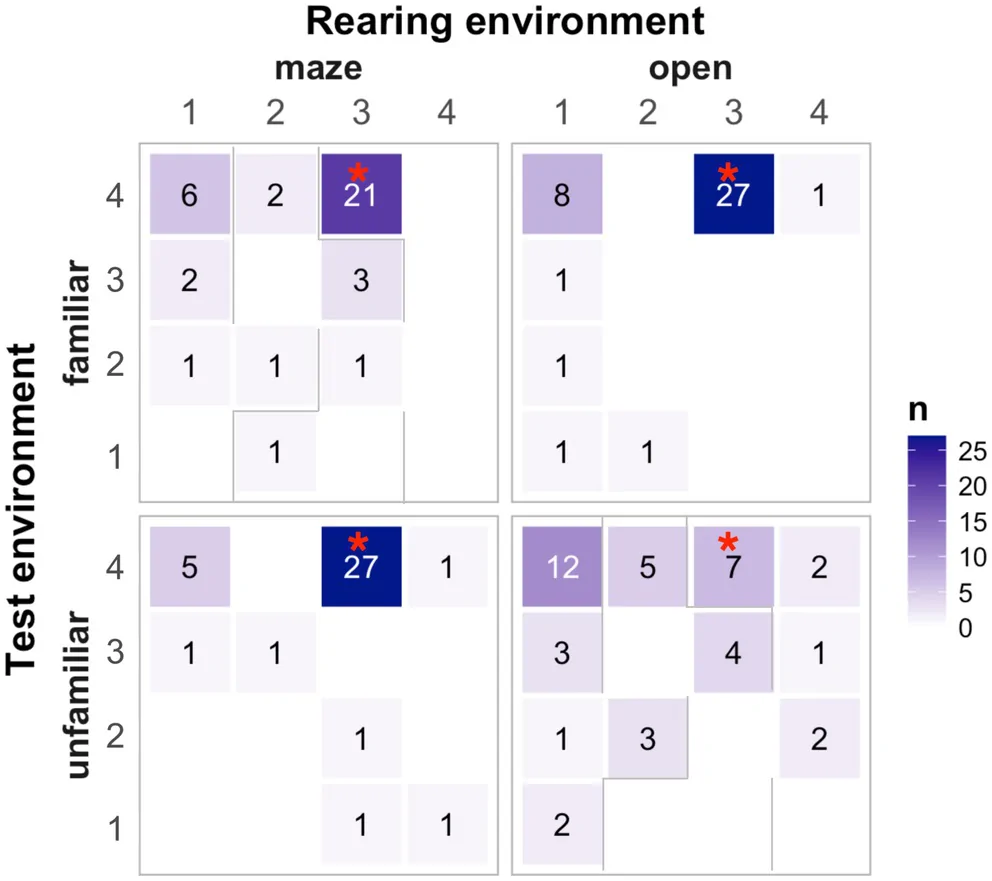
Heatmaps of individuals' final positions during the foraging assay, recorded using an analytical grid system. Panels show butterflies reared in maze and open environments (rearing environment), separated by familiar and unfamiliar test conditions (test environment). Values indicate the number of individuals occupying each grid cell at the end of the essay, and red asterisks mark the feeder location.

## 4 Discussion

Our results show that the benefits of prior experience on foraging efficiency depend on environmental complexity in *Heliconius erato phyllis*. Butterflies reared in a complex, maze‐like environment performed similarly when tested in their familiar maze and in the unfamiliar open environment, whereas butterflies reared in a simple, open environment performed substantially worse when tested in the unfamiliar maze. These findings indicate that prior experience of spatially complex environments provides greater benefits in spatially structured than in open environments, consistent with the predicted ecological benefits of spatial memory in a butterfly with well‐known ecological (Young and Montgomery 2020; Young, Melo‐Flórez, et al. 2024) and neuroanatomical (Montgomery et al. 2016; Couto et al. 2023; Sivinski 1989) specializations for learning. Given that *Heliconius* require pollen resources to maintain chemical defenses, somatic condition, and reproductive output (O'Brien et al. 2003; Dunlap‐Pianka 1979; Gilbert 1975; Jones 1930; Pinheiro de Castro et al. 2025; Foley, McPherson, Roger, Batista, et al. 2026), but must compete with other butterflies and hummingbirds for these resources (Gilbert 1972), increased foraging efficiency is likely to contribute to overall condition and fitness, particularly where competition for pollen resources is high (Murawski and Gilbert 1986).

The unusual reliance of *Heliconius* on pollen may therefore help explain why learning‐dependent foraging is particularly important in this group. Unlike other Lepidoptera, adult *Heliconius* repeatedly exploit spatially restricted pollen resources throughout their extended adult lives (Foley, McPherson, Roger, Batista, et al. 2026), which may span upwards of 6 months (Ehrlich and Gilbert 1973), providing repeated benefits from information acquired through experience. Where resources occur predictably enough to be revisited but vary in their spatial distribution, learning may allow individuals to efficiently exploit local sources while retaining the capacity to adjust their foraging behavior when conditions change. However, such flexibility is unlikely to be cost‐free. Neural tissue is energetically expensive to develop and maintain (Niven and Laughlin 2008), suggesting that investment in enlarged and plastic mushroom bodies may be favored where the fitness benefits associated with learning outweigh these costs.

Learning‐dependent foraging is particularly relevant in *Heliconius*, a genus that relies on spatial memory to establish and maintain traplining routes between feeding sites (Gilbert 1975; Mallet 1986). Efficient trapline behavior requires learning the locations of resources and may also require flexibility when spatial relationships change or following environmental disturbance. Although our experiment addressed the potential benefits of experience with spatial complexity, it did not directly test the ability to reorganize learned routes in a novel complex environment. Our findings also align more broadly with evidence from other insects indicating that more complex environments enhance cognitive performance in crickets (Mallory et al. 2016), flies (Díaz‐Fleischer et al. 2009) and bees (Boff et al. 2020). Here, we demonstrate that experience acquired in a spatially structured environment provides substantial benefits for subsequent navigation within that environment. We also observed a surprising general decline in task completion with age, with younger individuals performing better. However, this age‐related pattern did not interact with rearing environment, indicating that the influence of environmental complexity on foraging efficiency was robust across ages. Given that long‐term memory has been shown to remain robust with age over experimental periods longer than those examined here (Foley, McPherson, Roger, Young, et al. 2026), we suggest the age effect may instead reflect changes in motivation.

While our results demonstrate clear experience‐dependent effects of environmental complexity on spatial foraging, several limitations should be considered. Spatial complexity was manipulated solely through the addition of internal partitions, and we did not manipulate specific environmental components such as resource distribution, social interactions, or sensory variation. Rather, the maze environment specifically increased the navigational costs of locating the feeder by constraining direct flight paths, representing one ecologically relevant form of environmental complexity. In addition, due to space constraints, butterflies were not tested in a fully novel environment, limiting our ability to assess how experience in one complex environment might transfer to other complex environments, to examine if the experience of spatial complexity promotes increased behavioral plasticity, as noted in other systems (Brown et al. 2003; Rodewald et al. 2011; Clarin et al. 2013; Carducci and Jakob 2000; Díaz‐Fleischer et al. 2009). Importantly, however, distinguishing between these alternatives was not the primary objective of this study. Rather, our aim was to test whether previous spatial experience provides greater behavioral benefits under spatially structured than in simple environmental conditions. Finally, lower completion rates among butterflies from the open environment tested in the unfamiliar maze reduced sample sizes for some comparisons, and the results on foraging speed should therefore be interpreted with appropriate caution, although the lower completion rates are also consistent with predicted effects. We note that a similar experiment in *H. hecale* (Hebberecht et al. 2023), using open and maze environments but with a smaller sample size (*n* = 21), found that individuals reared in the more complex environment showed higher foraging efficiency in the maze environment than those reared in the open environment, consistent with our results. The consistency of these findings across experiments with similar designs, but using different *Heliconius* species ( *H. erato* and *H. hecale*) and conducted with different cage dimensions and in distinct localities, in Panama and northeastern Brazil, strengthens confidence that the observed effects are generalizable across *Heliconius*.

Together, our findings indicate that the benefits of learning‐dependent foraging strategies are greatest in more spatially complex environments in a nonsocial insect with strong ecological dependence on spatial learning. By showing that previous experience provides greater foraging benefits in spatially structured than in simplified environments, this study highlights environmental structure as a likely determinant of the ecological value of spatial learning. This conclusion is consistent with evidence that the benefits of memory depend on the richness and spatial distribution of available resources (Pull et al. 2022) and with theoretical predictions that memory‐guided and adaptive learning strategies provide greater foraging advantages in heterogeneous landscapes, provided that foragers can perceive and retain information about their local environment (Bracis et al. 2015; Gibbs et al. 2024). More broadly, these results suggest that habitat simplification may reduce the benefits of learning‐dependent foraging in wild insect populations by decreasing the spatial heterogeneity and range of foraging decisions over which learned information improves performance.

## Author Contributions

**P. A. Moura:** conceptualization (equal), data curation (lead), formal analysis (lead), investigation (lead), methodology (equal), project administration (equal), resources (lead), validation (equal), visualization (lead), writing – original draft (lead), writing – review and editing (equal). **M. Z. Cardoso:** conceptualization (equal), project administration (supporting), supervision (supporting), writing – review and editing (supporting). **S. H. Montgomery:** conceptualization (equal), funding acquisition (lead), methodology (equal), project administration (equal), supervision (lead), validation (equal), writing – review and editing (equal).

## Funding

This work was partially supported by the Coordenação de Aperfeiçoamento de Pessoal de Nível Superior—Brasil (CAPES)—Finance Code 001 to P.A.M. S.H.M. is supported by a NERC IRF (NE/N014936/1) and ERC Starter Grant (758508). M.Z.C. was supported by a Productivity grant (CNPq; Proc 476617/2011‐1 and 306985/2013‐6).

## Conflicts of Interest

The authors declare no conflicts of interest.

## Supporting information


**Figure S1:** Predicted probability of individuals reaching the feeder in the 5‐min navigation assay across age. Age was modeled as a continuous predictor. Lines show model‐predicted probabilities of task completion for individuals tested in the familiar and unfamiliar environments, separated by rearing environment. Shaded ribbons represent 95% confidence intervals. The open rearing environment denotes the enclosure without internal partitions, whereas the maze rearing environment denotes the enclosure with internal partitions. Familiar indicates individuals tested in their home environment, whereas unfamiliar indicates individuals tested in the alternative environment.

## Data Availability

The data that support the findings of this study are openly available in Figshare at https://doi.org/10.6084/m9.figshare.31424675.
