# Supplementary material for: Experience Improves Spatial Foraging Performance in a Complex Environment in Heliconius Butterflies
Source: Ecol Evol. 2026 Oct 27;16(11):e74541. doi: 10.1002/ece3.74541 (PMC13713222; doi:10.1002/ece3.74541)
Supplement: Supplementary file 1 [file ECE3-16-e74541-s001.docx]

**Supplementary Information**

**Figure S1. Predicted probability of individuals reaching the feeder in the 5-minute navigation assay across age.** Age was modelled as a continuous predictor. Lines show model-predicted probabilities of task completion for individuals tested in the familiar and unfamiliar environments, separated by rearing environment. Shaded ribbons represent 95% confidence intervals. The open rearing environment denotes the enclosure without internal partitions, whereas the maze rearing environment denotes the enclosure with internal partitions. Familiar indicates individuals tested in their home environment, whereas unfamiliar indicates individuals tested in the alternative environment.
